# Balancing on the redline: a qualitative study of the experiences purchasing sugar-sweetened beverages among Indigenous adults in a Canadian urban neighbourhood

**DOI:** 10.17269/s41997-023-00831-z

**Published:** 2023-11-30

**Authors:** Maria Kisselgoff, Michael Redhead Champagne, Riel Dubois, Lorna Turnbull, Jeff LaPlante, Annette Schultz, Andrea Bombak, Natalie Riediger

**Affiliations:** 1 Department of Food and Human Nutritional Sciences, University of Manitoba, Winnipeg, MB Canada; 2 Fearless R2W, Winnipeg, MB Canada; 3 Faculty of Law, University of Manitoba, Winnipeg, MB Canada; 4 National Indigenous Diabetes Association, Winnipeg, MB Canada; 5 College of Nursing, University of Manitoba, Winnipeg, MB Canada; 6 Department of Sociology, University of New Brunswick, Fredericton, New Brunswick Canada

**Keywords:** Sugar-sweetened beverage, Urban, Indigenous, Discrimination, Food desert, Food insecurity, Boissons sucrées, urbain, indigène, discrimination, désert alimentaire, insécurité alimentaire

## Abstract

**Objective:**

Growing evidence suggests that inner-city residents actively navigate their food landscape to meet a wide range of socio-economic needs. Given the increasing focus of health policies on sugar-sweetened beverages (SSB) through price-based strategies, it is critical to understand purchasing habits of populations with higher SSB intake. This study examined urban Indigenous adults’ SSB shopping behaviour and experiences.

**Methods:**

We conducted a community-based participatory research study using semi-structured interviews with a purposive sample of Indigenous adults (≥ 18 years old) from the North End neighbourhood of Winnipeg. Interviews were audio recorded, transcribed verbatim, and thematically analyzed.

**Results:**

All 20 participants (women = 10; men = 8; two-spirit = 2) consumed SSB on a regular, daily basis either at the time of the interview or at a prior period in their lives. Themes defining residents’ SSB shopping behaviour and experiences of shopping for SSB included *balancing on the redline*, specifically (1) *balancing SSB purchasing constraints and facilitators with savvy shopping approaches* and (2) *balancing (stereo)typical reactions with resilient coping approaches*. Residents procured SSB in various stores within and beyond the boundaries of the North End neighbourhood. SSB is a considerable, reoccurring expense, requiring savvy price-shopping strategies in order to access. Indigenous adults experience judgement and stereotyping when purchasing SSB, including intersecting racial, class, and weight stigma.

**Conclusion:**

Purchasing SSB is perceived as a source of judgement when outside of inner-city neighbourhoods. Policymakers should consider how policies directed at SSB, which are consumed by Indigenous and food-insecure populations in greater quantities, may magnify existing racial, class, and weight-based discrimination.

## Résumé

**Objectif:**

De l’évidence grandissante suggère que les résidents urbains naviguent activement leur environnement alimentaire pour rejoindre multiples niveaux socio-économiques. Étant donné l’intérêt grandissant envers nos principes de santé pour boissons sucrées (BS) en travers des stratégies à base de prix, il est critique de comprendre les habitudes d’achat des populations qui consomment le plus de BS. Cette étude a examiné les habitudes et expériences d’achats de BS d’adultes urbains Indigènes.

**Méthode:**

Nous avons fait une recherche participative dans une communauté utilisant des interviews semi-structurés utilisant un échantillon d’adultes Indigènes (≥ 18 ans) de la communauté « North End » à Winnipeg. Les interviews ont été enregistrés, transcrits verbatim et analysés thématiquement.

**Résultats:**

Tous les 20 participants (femmes = 10; hommes = 8, bispirituel = 2) ont consommé des BS régulièrement, à tous les jours, soit au temps de l’interview ou à un temps passé dans leur vie. Les thèmes qui définissent les habitudes d’achat de BS des résidents et leurs expériences d’achat de BS incluent : *balancer sur la ligne rouge*, en particulier (1) *balancer les contraintes et facilitateurs d’achat de BS avec des approches d’achats astucieux* et (2) *balancer les réactions (stéréo)typiques aves des approches d’adaptation résilientes*. Les résidents se procurent des BS dans multiples magasins à l’intérieur et à l’extérieur des limites de la communauté « North End ». Les BS sont une dépense considérable et récurrentes qui requiert des stratégies de prix d’achats astucieux. Les adultes indigènes expérience du jugement et des stéréotypes lors d’achats de BS, qui incluent des stigmatismes raciaux, classistes et à base de poids.

**Conclusion:**

L’achat de BS est perçu comme une source de jugement lorsqu’on sort des communautés du centre-ville. Les décideurs politiques devraient considérer que leur politique envers les BS, qui sont disproportionnellement consommés par les populations radicalisées et alimentairement insécures, peuvent aggraver les discriminations raciaux, classistes ou basées sur le poids.

## Introduction

Of growing public health concern is sugar-sweetened beverage (SSB) intake, due to associations with the development of type 2 diabetes (Malik et al., 2010). For the purposes of this study, SSB will specifically refer to drinks with added sugar; beverages containing naturally occurring sugars (e.g. 100% juice and milk) and diet beverages are not included. SSB have been the focus of proposed fiscal policy efforts aimed at deterring its purchase, namely via taxation, with Newfoundland and Labrador implementing a SSB tax in 2022 (Department of Finance Newfoundland and Labrador, 2022). In 2015, Indigenous^1^ peoples living off-reserve in Canada consumed significantly greater daily per capita volume (mL) and energy intake from SSB compared to other ethnic/racial groups, with a mean volume of 303 mL compared to, for example, white populations who reported a mean of 204 mL (Jones et al., 2019); populations with lower income and education, as well as experiencing food insecurity also report higher SSB intake (Warren et al., 2022). As such, proposed SSB taxation policies implicitly target both lower income and Indigenous populations. The off-reserve context is of particular importance given that over half of the Indigenous population in Canada lives in urban centres, with the proportion predicted to continue increasing (Anderson, 2019). Therefore, it is imperative we explore the factors and experiences that underline urban Indigenous people’s procurement of SSB in the context of proposed and newly implemented taxation policies.

Patterns of SSB intake and purchasing among Indigenous populations are shaped by a variety of structural and social determinants that correspond to the legacy of settler colonialism. Settler colonialism operates across three inter-related pillars including power structures (e.g. legal, political, and economic systems), spatial structures (e.g. food environments, segregation, reserve systems), and social narratives (e.g. prevailing stereotypes and racism) that permeate the lived experiences of Indigenous people (Barker et al., n.d; Smith, 2021). These determinants shape where and how Indigenous people live, work, access food, and feed their families; and how settlers benefit from the lands and their products (e.g. sugar).

Power structures influence SSB intake through economic means. Food insecurity among urban Indigenous adults is approximately three times higher compared to among non-Indigenous households (Tarasuk et al., 2014). Food insecurity is defined as “inadequate or uncertain access to food because of financial constraints” (Health Canada, 2017, p 13). However, an analysis of overall diet quality among Indigenous adults, as compared with non-Indigenous adults, revealed that disparities in diet quality persisted after adjustment for food security (Riediger et al., 2022), highlighting the need for a more comprehensive investigation into the determinants that shape Indigenous peoples’ present-day foodways.

Spatial structures may influence SSB intake via access and availability. Urban Indigenous people in Canada are more likely to live in low-income, subsidized housing, to rent rather than own, and to live in neighbourhoods separate from non-Indigenous people (Anderson, 2019). As such, Indigenous people are more likely to live in areas known as grocery redlined regions, sometimes referred to as food deserts, and food swamps. Redlining describes policies and practices that diminish opportunities for residential investment in largely racialized neighbourhoods, and contribute to cycles of neighbourhood decline (Eisenhauer, 2001). Grocery redlined areas are characterized by a higher concentration of corner stores but a lack of full-service grocery stores with access to fresh and nutritious foods (Eisenhauer, 2001; Vilar-Compte et al., 2021). These areas are also prone to having food swamps, defined as areas where healthy food options might be available, but they are overshadowed by an overwhelming abundance of high-calorie foods and beverages options, such as fast-food outlets (Vilar-Compte et al., 2021). There remains academic debate about the most appropriate terms to describe similar social phenomena. The term food desert, for example, has been critiqued as implying it is a natural phenomenon (e.g. desert) as opposed to socially constructed (Gripper et al., 2022); for this reason, many activists and scholars prefer the term food apartheid (e.g. Gripper et al., 2022). The term “redlined” resonated with community partners in Winnipeg for the present study. Winnipeg, Manitoba has the largest Indigenous population (by number) of any metropolitan area in Canada, constituting approximately 12% of the urban population (City of Winnipeg, 2018). However, in the North End (NE) neighbourhood of Winnipeg, 29% of residents self-identify as Indigenous (WRHA, 2019). The neighbourhood is further characterized as a food desert, where low-income areas are located > 500 m from full service or national chain food stores (Manitoba Collaborative Data Portal, updated Oct 28, 2021).

Overt racism continues to permeate Indigenous people’s lives in Canada, with documented discrimination in the Canadian urban context (e.g. Canel-Çınarbaş & Yohani, 2019). How racism and social narratives pertaining to SSB and procurement may impact experiences of Indigenous adults have not been empirically explored. However, the racialization of weight, the geneticization of diabetes (Poudrier, 2007), and documented concern regarding weight stigma among Indigenous peoples (Cyr & Riediger, 2021) are products of the settler colonial legacy that may inform related narratives regarding SSB, weight, and diabetes. Growing lines of inquiry suggest that in-store consumer shopping experiences are key determinants shaping food-purchasing patterns and ultimately consumption (Cannuscio et al., 2014). Studies report that social interactions, mediated by patrons’ socio-economic status (Cannuscio et al., 2014) and race (Pittman, 2020; Zenk et al., 2014), have considerable influence on the location and store type that people choose to shop in. Negative interactions with staff and fellow shoppers reduce consumers’ likelihood of returning to certain stores (Cannuscio et al., 2014). These experiences further influence access to certain foods and subsequently diets, given that the quality and variety of food differs between types and locations of stores (Cannuscio et al., 2014).

Retail food establishments are targeted sites of intervention for proposed SSB tax policies; however, to our knowledge there have been no studies assessing the in-store customer experiences of Indigenous people shopping for SSB. This research is especially relevant given the current policy conversation in Canada around the proposed taxation of SSB, and its first implementation in the province of Newfoundland and Labrador (Department of Finance Newfoundland and Labrador, 2022). It is critical to investigate how and what drives consumption and shopping behaviours before control measures are implemented, especially those directed at Indigenous populations who have been, and continue to be, harmed by colonial policies. Exploring the point of intervention will allow for facilitation of policies that consider the factors that affect food patterns and expand on specific predictors of SSB purchasing. Therefore, the objectives of this study were to (1) explore the structural and social determinants influencing purchasing of SSB and (2) explore the experiences of urban Indigenous adults purchasing SSB.

## Methods

### Design

This community-based participatory research study (Minkler et al., 2008) was conducted in partnership with the National Indigenous Diabetes Association (NIDA), an Indigenous organization focused on diabetes prevention and management, and Fearless R2W, an Indigenous-led community non-profit in Winnipeg’s North End (NE) focused on child welfare. Concerned for the implications of an SSB taxation policy on a community largely situated within a food desert and affected by high prevalence of food insecurity, we set out to explore the NE residents’ lived experience of purchasing SSB.

Our study is part of a larger research project exploring the acceptability of a proposed SSB tax among Indigenous populations in Manitoba. Formal research agreements were signed with Indigenous community partners outlining the values underpinning our partnership, each party’s responsibilities, as well as access and ownership of the data. Other co-authors identify as white settler women, and have diverse grocery retail experiences, class backgrounds, and weight trajectories. Their academic expertise in public health, nutrition, law, nursing, and sociology have also influenced their critical perspectives of health policies. In keeping with the study design, we engaged reflexively throughout the study.

Study results were previously shared with the broader study team, which included an Elder, at a gathering to discuss study findings. Results were also shared with the NE community at an in-person event with Fearless R2W. Attendees had the opportunity to discuss study findings and contribute to interpretation. Finally, participants also received several infographic summaries of study findings, and were invited to provide feedback on each.

### Decolonizing framework

We adopted a decolonizing framework that involves actively seeking out and acknowledging colonial aspects of policies and dismantling them by centering Indigenous perspectives to challenge the corresponding spatial, power, and social constructs (Barker and Lowman, n.d.; Smith, 2021). We specifically analyzed how power structures, spatial structures and social narratives *interacted* to influence the experiences of SSB procurement for Indigenous adults.

### Setting

The NE community area is located centrally in Winnipeg, Manitoba, on Treaty One Territory. The majority of Indigenous people in Winnipeg identify as Métis (54%) or First Nations (44%) (City of Winnipeg, 2018). The Indigenous population is a young and fast-growing demographic, with 46% aged 24 years and under (City of Winnipeg, 2018). Residents of the NE experience greater social and health disparities compared to the rest of Winnipeg, including higher unemployment rates (9.5% vs. 6.5%), diabetes prevalence (12.1% vs. 7.9%) (WRHA, 2019) and percentage of adults labeled “overweight” or “obese” according to the Body Mass Index (65% vs. 54%) (WRHA, 2015).

### Participants and recruitment

We aimed to recruit Indigenous adults residing in or near Winnipeg’s NE neighbourhood. Eligibility criteria included (1) self-identify as Indigenous (First Nations, Métis, or Inuit), (2) 18 years of age or older, (3) comfortable speaking English, (4) self-identify as residing in the NE, and (5) able to attend a 1-h interview. We utilized purposive sampling to recruit participants who self-identified as regular SSB consumers, food insecure, and parents or guardians. We also sought the perspective and knowledge of Elders with strong connections to the NE Indigenous community as interview participants. A community research assistant (third author) was hired to ensure reciprocal transfer of knowledge and skills, and to lead the recruitment process. The community research assistant also assisted with revising the interview guide, interpretation of data, and knowledge mobilization.

### Data collection

We conducted in-depth, semi-structured interviews following an interview guide, which was updated iteratively to capitalize on emerging issues. Participants also completed a demographic questionnaire to describe sample characteristics. We completed interviews between November 2019 and August 2020, with the first six interviews conducted face-to-face by the first author in a private room at the institution’s Inner-City Social Work Campus, located in the NE. Due to COVID-19 social-distancing orders, the remaining interviews were moved to over the phone. All interviews were audio recorded and transcribed verbatim. Following each interview, researchers debriefed and recorded field notes. One-on-one or two-on-one phone interviews with participants were conducted by the first author and/or the Principal Investigator. Before beginning interviews, it was clarified that our research group is conducting the research in partnership with two Indigenous-led organizations. All participants provided their individual informed consent and received an honorarium.

### Analysis

We coded and analyzed transcripts in NVivo 12, following Braun and Clarke’s (2006) theoretical thematic approach. We analyzed the data with prior engagement with the literature and the application of the decolonizing theoretical framework. Following active and repeated reading of the transcripts, we generated descriptive codes, which were later grouped into themes. Descriptive coding involves synthesizing the meaning within each relevant data extract into words or short phrases (Saldaña, 2011). We paid particular attention to the language and tone used by the participants relating to the three main components of settler colonialism in the context of SSB procurement and food environment. By doing so, we bring attention to the extent that power, spatial, and social norms influence the experiences and types of stores, foods, and SSB that are regularly accessible to the residents of the NE financially, geographically, and socially.

The community research assistant and research partner member-checked and analyzed data together with the lead author. Both the community research assistant and partner share a similar context to the participants, as long-term residents of the NE community who self-identify as Indigenous. Through the member-checking process, they facilitated an accurate interpretation of what the participants communicated, as well as the soundness of the codes and themes generated (Creswell & Miller, 2000 p. 127). Results were also presented to the community partner, Fearless R2W, through a community event to seek out further feedback and through newsletters published by the NIDA.

## Results

### Participant characteristics

We interviewed 20 participants (10 women, 8 men, and 2 two-spirit) with an average interview length of 48 min (range 25–94 min). The age of participants ranged from 20 to 65. The sample closely represents the Indigenous population in the NE, with 75% self-identifying as First Nation, 20% as Métis, and one participant as “Indigenous”. At the time of the interview, 15 participants were not working, four were employed part-time, and one was employed full-time. Most participants had completed high school (*n* = 11) and four had a college education. When completing the demographic form, 60% reported consuming SSB daily; however, during the interview, it was determined that 85% (*n* = 17) of participants consumed a SSB at least once daily. The three participants who did not consume SSB reported drinking diet beverages. All participants reported consuming SSB on a regular, daily basis at some point in their lives. Most participants rated their health as good, very good, or excellent (85%).

### Thematic analysis

The experience of purchasing SSB among Indigenous adults residing in the NE, an inner-city community area, is defined by the overarching theme, *balancing on the redline*. This theme captures the balancing act residents of the NE engage in when shopping for SSB, having to navigate the financial, social, and geographical challenges within and beyond the boundaries of their neighbourhood.

We identified two sub-themes illustrating the experience of *balancing on the redline*. The first sub-theme “balancing SSB purchasing constraints and facilitators with savvy shopping approaches” captures the intersecting spatial and economic determinants that shape the NE residents’ SSB shopping patterns, and the resourceful strategies residents employ to afford and acquire SSB. The second sub-theme “balancing (stereo)typical reactions with resilient coping approaches” describes the social aspect of shopping in-store for SSB, defined by types of reactions participants received, as well as how they felt and coped with them. Table 1 summarizes these sub-themes and corresponding categories with exemplary quotes using pseudonyms.

**Table 1:** Summary of sub-themes and categories with corresponding exemplary quotes

Theme	Sub-themes	Categories	Quote
Balancing SSB purchasing constraints and facilitators with savvy shopping approaches	Facilitators	Food landscape is redlined	“Like if you go to, uh, 7-Eleven or, uh, another store, they jack the the price up to, uh. But if you go to like, uh, Shoppers or stuff, other pla- uh, uh, a pharmacy or type place they are sorta on sale, uh, like 3.99 or 3.50 vs that. And I think, I don’t know if there’s a- a- a expiry date on these why they put them on sale.” (Alan) “Oh, again, I guess the lack of grocery stores and the fact that people have to go to corner stores and it’s … corner stores, the healthy food is like three times more expensive than it is at the grocery store…Specifically for me and my family it’s mostly um, the healthy foods because uh, like I- like I- like I said, my husband and I are both diabetic, so it’s kind of, um, really hard for us to get the healthy foods that we need. We don’t drive. So, we have to walk or take a bus or rely on cabs and make the once in a while friend to pick us up at the grocery store.” (Janice)
		SSB deals and discounts	“You see those little deals. Okay, this one has a better deal than the other store, so maybe I’ll go to this store. … I know some places it [a canned drink] costs a dollar, whereas it… that place is like cheap to get a whole bunch of canned drinks from that store, and also even like two liters. That’s the cheapest place I would say to get pop.” (Gerry)
		Relative price of SSB	“So that’s why you see the kids around here eating big bags of chips and eating bags of candy and pop because it’s cheaper than anything else. It’s what we’re able to afford.” (Louise)
	Constraints	Tight budgets	“Usually when I don’t have enough to buy um, a case of pop I would buy three or four to last me through the day.” (Shelly)
		SSB is both cheap and unaffordable	“I just asked them like, ‘Can you help me? Like hook it up with a drink, I don’t have any pop.’ Or I just go look for some. ‘I need change for some pop.’ So yeah.” (Carolyn)
	Savvy shopping	Price shopping	“You see those little deals. Okay, this one has a better deal than the other store, so maybe I’ll go to this store. … I know some places it [a canned drink] costs a dollar, whereas it… that place is like cheap to get a whole bunch of canned drinks from that store, and also even like two liters. That’s the cheapest place I would say to get pop.” (Gerry)
		Heightened awareness	“When I, okay well say if I go to Walmart or like a superstore or something, I’ll buy my Gatorade there, because it’s, uh, a little bit cheaper than going to the corner store, definitely.” (Raymond)
		Stretching	“But I don’t really buy pop that often. Uh, it’s more so, it’s just juice because it goes… it stretches farther. Like, I can, you know, buy that one bag o’ juice and I can make three big jugs out of it. So that’s gonna last me a few days, opposed to if I bought, uh, a two liter pop that’s gone in, you know, 10 min. You get four cups or whatever out of it. Whereas the juice, like, it stretches and goes farther.” (Louise)
		Sharing is caring	“Cause I mean I think about my family too … It’s like if I buy myself a pop, I’m gonna obviously just get two-liters instead.” (Tyler)
Balancing (stereo)typical reactions with resilient coping approaches	Judgement-free zone	Regulars	“they’re like oh hey! You’ve come back again! Uh …they know me pretty much, because I’ve been going there since I was little.” (Shelly)
		No reaction	“Um, well, I don’t think I get a reaction at all because it’s such a, such a normal thing for somebody to go to the store and buy some sort of beverage.” (Diane)
	Danger zone	SSB judgement	“I know one person was like, ‘What do you need all those drinks for?’ And I’m like, ‘To last me all month.’ But sometimes, like say if someone gives me a haircut or if she cuts my hair, one two four is for her and her kids, right? So it’s usually like I’ll have a pack for myself, but I notice I drink, yeah, they always ask me, ‘You need all that pop?’” (Carolyn)
		SSB judgement + racism + weight	“my weights always been a problem. Since I was a little girl my weight fluctuated. And so, um, I’m used to people looking at me with this look of why are you buying sugar when you’re already that big, you don’t need it type of look…Um, so, uh, it kind of- it felt like, um, I was being judged because of my weight and because I’m- I’m First Nation.” (Janice)
		Racism profiling theft	“like when I am by myself, not so much. But when I am there with my son [name], oh yea! people are so strongly opinionated about kids, its crazy. So my son is 13, and now when he goes to the corner store he’s like ‘mom I need something with shorter sleeves, because when I have things that are too long, then I get checked at the door’. He is only 13, and for him to have experiences like that and be normalized it is kinda freaky.” (Amanda)
		SSB judgement + racism	“The- the clerk, my wife went in there and she bought a Pepsi, and she bought a bag of chips for me, and she was going through the line, and then the clerk like asked her, uh, ‘Who you buying these for?’ And, um, my wife said, ‘My husband,’ and then the clerk said, ‘Is your husband, uh, the native guy, the big native guy with tattoos on his neck?’ And so my wife laughed and said like, ‘Yeah, that’s, uh, that’s my husband,’ and then sh- they were laughing and the clerk was like, ‘You know, I know, because he comes in here and he buys the same, he buys the same stuff.’” (Raymond)
	Coping mechanisms	Armouring up	“Um, if they do, I don’t notice. I don’t really actually pay attention. I don’t pay attention. Um, I have no space in my life or head space for, um, ignorance or any of that. And so, I find this past year I’ve been, um, paying less attention to that kind of stuff when I’m out in those spaces.” (Louise)
		Reframing	“Um, no. Even if I did, I don’t think that I would, like, pay attention, because, it’s, like, if somebody has a problem with what I’m buying that’s their problem, because they’re not the ones that are eating it, or drinking it, so, yeah. I don’t really pay attention to, like, people and their reactions on things because I know what I’m doing, or, you know what I mean? So, like, they need to pay attention to themself.” (Evelyn)
		Regulating	“well, my son and I have big talks about his experience here because he does not always understand why things are hard for him. Even though everywhere else I take him, it is not hard. The only things that are hard for him are school, and some of the interactions with the neighbours behind the mall area. But um, when I was younger it used to make me feel like I was that ‘dirty Indian’, now that I am older and I feel people judging me, then its just for me, its more a reflection of themselves. That them judging me means that they have enough problems in their life to feel like they could take up some more, you know [laughs]. So like I’m glad they wasted their energy [laughs].” (Amanda)

### Balancing SSB purchasing constraints and facilitators with savvy shopping approaches

Participants’ SSB shopping habits are shaped by the food options (un)available to them, their personal, family, and community income level, and the strategies they use to balance these barriers and facilitators. The food environment of the NE is characterized by participants as what scholars would refer to as a food desert or redlined; corner stores are extremely abundant while grocery stores are sparse and often not within walking distance. Available food is regularly in poor nutritional quality, freshness, and variety, and is marked up in price. SSB is among many foods participants describe as not healthy but readily available to them.

For those that just stay in the area, like obviously just the easy chips, noodles, Pepsi, and just the bread that they need, and everything else that goes with it. When impoverished you have X amount of things you can actually get. (Gavin)

Participants described SSB and other foods they consume not just as unhealthy, but also as discursively linked to being lower class.

We are living off of scraps of society’s leftover from their plates. That’s what... We take only what we can get. Um, which isn’t, uh, good for us. You know, like, we don’t get healthy options food-wise, and when we do, we can’t afford it. (Louise)

A few participants pointed out that food-purchasing patterns in the NE have been conditioned by the food environment over time. One participant suggested that even if presented with novel food options, NE residents will default to the familiar. SSB shopping patterns were also influenced by their perception of SSB affordability, which was mediated by their personal financial means, the relative price of SSB to other food products, as well as price discounts. SSBs were described in contrasting terms, even among the same participants, as both “cheap” and “expensive” over the course of their interview, and were often still out of reach financially. For example, in comparison to other foods, Louise described pop to be “cheaper than anything else”, while describing Slurpees (a partially frozen pop beverage) to be a “big splurge” for her personally.

Some participants described instances in which they could not afford to buy SSB or a specific quantity of SSB they wanted to buy, particularly bulk because of cheaper price per unit. As a result, those tight for cash end up purchasing single cans, ultimately paying more for SSB over time. Among the participants who discussed SSB cost at different store types, most indicated that SSBs were priced higher in corner stores than in full-service grocery stores. Store SSB pricing practices were described by some participants as influencing how they, or others, perceive the affordability of SSB, and ultimately shopping patterns. Participants indicated that SSBs were, more often than not, sold as part of a “deal” or “sale” rather than at regular price. Therefore, SSB deals influenced participants’ perceptions of SSB affordability, where they chose to shop for SSB, and how much SSB they bought at once. When describing SSB sold as a deal, participants referred to SSB as being more affordable, incentivizing them to buy more.

If there’s a deal for two liters of Pepsi or Coke Zero I’ll- I’ll buy the two for what, uh, two for five or whatever, kind of, thing. (Evelyn)

Participants also described how SSB pricing fluctuates, with deals often varying between different stores and changing often. A couple of participants raised questions regarding the intent behind deals on SSB.

Like if you go to, uh, 7-Eleven or, uh, another store, they jack the the price up to, uh. But if you go to like, uh, Shoppers or stuff, other pla- uh, uh, a pharmacy or type place they are sorta on sale, uh, like 3.99 or 3.50 vs that. And I think, I don’t know if there’s a- a- a expiry date on these why they put them on sale. (Alan)

Many participants described the strategies they employ as savvy shoppers to afford and share SSBs with family and friends, who may also be facing food insecurity. Some participants also discussed being the recipient of SSB from community members or family sharing. An undercurrent of this strategy was participants’ heightened awareness of SSB prices and deals at multiple different stores, often down to the cent as Carolyn mentioned one store selling Rockstars for “$5.63”.

Being equipped with data on SSB pricing, deals, and stores, participants described how they actively sought out a better price on SSBs. While discounts on SSB may prompt a purchase or a larger purchase of SSB on the spot, participants also dedicated significant efforts to pre-emptively price shop at certain stores, looking for discounts, deals, value packs, and 2 L bottles, as well as brand versus no-name SSBs. A few participants described buying SSB products that can be “stretched” such as chocolate syrup and or fruit drink concentrate (e.g. frozen or powdered drinks added to water) as opposed to pop, as Louise described, juice “gonna last me a few days, opposed to if I bought, uh, a two liter pop that’s gone in, you know, 10 min”.

Another strategy to cope with the shared experience of food and financial insecurity among NE residents involved buying enough SSB to share with other family members and friends. This gesture was described as an unspoken act of care that was expected of everyone. Many participants indicated that when buying SSBs, they would often buy more to share with others or others would share with them.

### Balancing (stereo)typical reactions with resilient coping approaches

Participants described the typical reactions they received while purchasing SSBs and when negative, the various strategies they used to cope with them. The nature of the reactions was mediated by the type, location, and context of the store and quantity of SSB purchased, as well as participants’ individual characteristics. Stores located within and in the proximity of the NE boundaries were generally regarded as safe shopping spaces. Many participants reported receiving virtually no reactions when buying SSB.

Participants who typically bought SSB at corner stores within the neighbourhood described forming friendly relationships with store owners and staff. When asked if anybody reacted to her drinking or buying SSB, Shelly answered “No, because they already know my routine”. She also went on to say that “they’re like oh hey! You’ve come back again! Uh …they know me pretty much, because I’ve been going there since I was little”.

Given the social norms underlying SSB consumption in the community, including the frequency of buying SSB at corner stores that carried and sold a lot of pop, a few participants were surprised when we posed the question about reactions to buying SSB. To many participants, buying SSBs was a normal routine, and some questioned why anybody would react in the first place.

I don’t think I get a reaction at all because it’s such a, such a normal thing for somebody to go to the store and buy some sort of beverage. (Diane)

A few indicated that the lack of reactions by convenience store workers was in the best interest of the business, given that SSB was an integral part of the cash flow, and the convenience stores are privately owned.

In contrast, some participants described shopping outside of the NE and/or in large chain grocery stores as unpleasant. Participants described receiving looks and comments questioning the contents of their shopping carts, specifically by white people. The reactions were described by participants as typical, something they expected when shopping because of what they were buying, their race, and/or their weight. For example, Carolyn was regularly questioned when buying SSB in “large” quantities by cashiers asking, “what do you need all those drinks for?” and “You need all that pop?”.

Two participants also indicated that judgement around SSB was amplified by weight stigma and racism. The two participants who described instances of weight stigma both identified as women and when the person identified as reacting was gendered, it was a woman. When describing reactions she received, Janice said “I’m used to people looking at me with this look of why are you buying sugar when you’re already that big, you don’t need it type of look… it felt like, um, I was being judged because of my weight and because I’m- I’m First Nation”. Beverly also described receiving negative reactions even when not shopping for SSB or other “junk” food items.

they look too ’cause ... Like, for me, I am overweight… it’s mostly like white, old ladies that look at me and just glance, just like snub me for like having junk food and pop… I don’t know. I feel like people have judgment toward other people of what they, what they eat or drink. They’re curious to know ... Maybe because they’re racist and they’re judging.

When shopping outside the NE, some participants described being regularly racially profiled as suspected thieves and were followed by security or checked at the door. The boundaries of the neighbourhood are not clear cut; however, it seemed the further away participants went, the reaction intensified.

when I’m in the North End, even at Giant Tiger here on North Main, or the Safeway down here on McGregor and Mountain, I don’t get followed. I don’t get stared at. I don’t get people watching me to make sure I’m not stealing. But when I go to a store outside the North End that happens every time, like clockwork. It’s like I expect it. (Janice)

One participant who shopped for SSB at a corner store outside the NE described feeling judged when the cashier identified him by the foods he regularly bought and that it was linked with his ethnicity describing him to his wife as “is your husband, uh, the native guy… I know because he comes in here… and he buys the same stuff”.

In order to balance the judgemental reaction participants received while shopping for SSB primarily outside of the NE boundaries, residents employed various protective, self-regulating, and reframing coping techniques. The first strategy was of “armouring up” in anticipation of reactions when shopping for SSBs in unsafe spaces. Armouring up took the form of intentionally, proactively blocking out reactions, or having a prepared come-back to questioning their purchases or intended purchases. One participant described having a prepared response in anticipation of judgement when purchasing “large” SSB quantities at once. Although those who completely blocked out reactions also tended to indicate that they did not receive reactions, it was implied that they have experienced stereotypical reactions in the past and/or that these continue to take place today.

Some coped with negative reactions by actively reframing the messages they received as a reflection of the reactor, and consequently an issue that is outside of the participant’s control. When asked about the impact the reactions had on participants, most described blocking and shrugging the negativity off to not allow judgemental reactions to lead to long-term effects and internalization of negative emotions. Underlying most coping mechanisms discussed by participants was a sense of self-trust and awareness, strengthening the armour against stereotypes and negative reactions around SSBs. Participants described knowing and trusting in the shopping choices that they made, making outside reactions irrelevant and ill informed.

In instances where reactions broke though the armour and caused distress, participants described how they persevered and took back control of their emotions. Participants discussed actively giving and seeking social support in response to stereotypical reactions. In response to being suspected as a shoplifter, Amanda described how they prepared their son to deal with shopping experiences.

## Discussion

SSBs are a regularly purchased and consumed product among the Indigenous community in the NE. Participants discussed how they proactively navigated their limited budgets, food environment, and cost of SSB, as well as potentially strained in-store interactions when procuring SSB. Experiences purchasing SSB involved an emotional process to actively balance these constraints and facilitators. Some of these aspects encourage the purchase of SSB while others challenge or shape access in different ways. Considerable thought went into SSB procurement; participants expressed preferences for the size of SSB as well as the type, location, and particular stores where they shopped for SSB, most often to maximize value for money, transportation, and familiarity. Importantly, these findings echo the notion that food and SSB purchasing practices extend beyond the relatively lower cost and/or abundance of SSB in the consumer’s immediate environment (Cannuscio et al., 2014). When presented with “healthful” food options, some participants continued to purchase what is familiar and comforting, and easily accessible. Existing evidence shows that food choices are conditioned by a variety of factors and may remain resistant to external forces for prolonged periods (Kelder et al., 1994). Therefore, SSB taxation policy neglects to account for the multi-dimensional process of food shopping practices and preferences, particularly among lower-income and Indigenous groups in an inner-city context. Policy makers should consider the long-term, conditioning aspects of determinants to food access when designing consumer-level interventions.

While SSBs were considered by some participants as a relatively inexpensive product, SSBs were also simultaneously described as unaffordable and represented a substantial financial expense for participants, many of whom were receiving social assistance. Despite having constrained economic means, residents continued to prioritize purchasing SSB for a variety of reasons, including the sharing and gifting aspect of purchasing SSB, which aligns with core Indigenous values of reciprocity.

Our findings also suggest purchasing SSB is a source of overt judgement intersecting with racism and weight stigma directed at Indigenous people, and largely perceived as enacted by non-Indigenous, white people. These findings are consistent with previous research from our team showing considerable judgement for SSB consumption, particularly directed toward individuals of higher weight and parents (Bombak et al., 2021; Waugh, 2022). The moralization of SSB purchasing and consumption was particularly intense among a highly educated, middle-class, white sample from another Winnipeg neighbourhood (Waugh, 2022). This moralizing undercurrent was also present in a recent response to a letter to the editor in the *Canadian Journal of Public Health* insisting advocating for SSB taxation is “altruistic” (Veugelers et al., 2022). We caution against moralizing a behaviour shaped by socio-economic circumstances and colonialism, as this moralization has the capacity to contribute to and compound structural and interpersonal stigma toward Indigenous people by taking away needed income (i.e. via taxation). Notably, all the participants were aware of the health implications of consuming SSB.

Participants’ experiences of being subjected to racial profiling and being suspected of being thieves echoes previous research with Indigenous youth in Winnipeg (Skinner & Masuda, 2013) and evidence among racial minority groups in the United States reporting discriminatory treatment when shopping in retail settings (Pittman, 2020; Zenk et al., 2014). Previous research has demonstrated that negative social interactions deter consumers from shopping in certain stores, which perpetuates social stratification and segregation (Cannuscio et al., 2014). This has strong implications for lower-income, racial minorities, given that they tend to live near to and conduct their primary grocery shopping in stores carrying predominantly less nutritious foods (e.g. Vilar-Compte et al., 2021). Findings that in-store experiences were more negative further from the NE are also consistent with research that found greater incidence of discrimination with a longer distance travelled away from the individual’s neighbourhood (Zenk et al., 2014), as well as qualitative research with Indigenous youth in Winnipeg (Skinner & Masuda, 2013).

To our knowledge, this study is the first to document the experience of urban Indigenous adults procuring SSB in the context of an inner-city Canadian neighbourhood. Our results offer insight into a consumer-level phenomenon that is heavily targeted by health policy, yet is understudied. Of note is the impact of the COVID-19 pandemic on our study, particularly on the data collection methods and connection with broader community and research partners. While the quality and trustworthiness of the findings were not compromised, the research process was significantly altered. Prior to the start of COVID-19, recruitment and interviewing were conducted in-person, and we regularly met with community research partners and the community research assistant. Once COVID-19 restrictions came into effect, all research was moved to a virtual format, with less opportunity to connect and share knowledge on a consistent basis, and community engagement is best conducted face-to-face.

## Conclusion

The experience of shopping for SSB for Indigenous people living in the NE is characterized by a delicate balancing act to manage their strained financial situation and risk of being stereotyped outside the community, where prices may be lower. Experiences are shaped by existing colonial structures. Power structures manifested as product prices and limited incomes. Spatial structures are represented by location of stores, physical availability, and lack of transportation limiting access. Finally, social narratives are marked by dominant discourses about food, health, and bodies, as well as Indigenous stereotypes. Importantly, these structures are intersecting and reinforce one another. For example, stereotypes and sense of stigmatization (i.e. social narratives) can discourage individuals from shopping outside their community (i.e. spatial structures), reinforcing existing segregation. This limited physical and social access (i.e. spatial structures intersecting with social narratives) to more affordable and healthier foods (i.e. economic power structures) in neighbouring communities further drives reliance on corner stores for SSB purchases. The intersections are important because it suggests that policies or programs addressing structural barriers (e.g. food prices or stereotypes) in isolation are unlikely to be effective without considering the entire colonial context. Health policies targeting SSB through taxation are likely to exacerbate existing colonial structures negatively impacting the health and well-being of urban Indigenous populations. Further research is needed to explore participants’ perspectives regarding taxation of SSB given the documented experiences and challenges described in the present study.

## Contributions to knowledge

What does this study add to existing knowledge?

- The study provides an in-depth description of the experiences of Indigenous adults living in an inner-city context in purchasing sugar-sweetened beverages.
- Indigenous adults report experiencing racism, weight, and poverty stigma when purchasing sugar-sweetened beverages, particularly when shopping outside their inner-city neighbourhood.

What are the key implications for public health interventions, practice, or policy?

- Findings have important implications for sugar-sweetened beverage taxation policies and health equity, particularly for Indigenous populations who are targeted by such a policy.

## Data Availability

Not available.

## Footnotes

1 Indigenous Peoples include First Nations, Métis, and Inuit, as defined in Section 35 of the Canadian Constitution of 1982.

## Code availability

Not available.

## References

[CR1] Anderson, T. (2019). Results from the 2016 Census: Housing, income and residential dissimilarity among Indigenous people in Canadian cities. https://epe.lac-bac.gc.ca/100/201/301/weekly_acquisitions_list-ef/2019/19-50/publications.gc.ca/collections/collection_2019/statcan/75-006-x/75-006-2019-18-eng.pdf

[CR2] Barker, A., & Lowman, E. B. (n.d.). Settler colonialism. *Global Social Theory*. https://globalsocialtheory.org/concepts/settler-colonialism/. Accessed Dec 2022.

[CR3] Bombak AE, Colotti T, Riediger ND, Raji D, Eckhart N (2021). Fizzy foibles: Examining attitudes toward sugar-sweetened beverages in Michigan. Critical Public Health.

[CR4] Braun V, Clarke V (2006). Using thematic analysis in psychology. Qualitative Research in Psychology.

[CR5] Canel-Çınarbaş D, Yohani S (2019). Indigenous Canadian university students’ experiences of microaggressions. International Journal for the Advancement of Counselling.

[CR6] Cannuscio, C. C., Hillier, A., Karpyn, A., & Glanz, K. (2014). The social dynamics of healthy food shopping and store choice in an urban environment. *Social Science & Medicine*, *122*, 13-20. https://doi-org.uml.idm.oclc.org/10.1016/j.socscimed.2014.10.005 10.1016/j.socscimed.2014.10.005 25441313

[CR7] City of Winnipeg. (2018). *City of Winnipeg Indigenous peoples highlights: 2016 Canadian Census*. https://winnipeg.ca/cao/pdfs/IndigenousPeople-WinnipegStatistics.pdf. Accessed Dec 2022.

[CR8] Creswell, J. W., & Miller, D. L. (2000). Determining validity in qualitative inquiry. *Theory into practice, 39*(3), 124–130. https://www.jstor.org/stable/1477543

[CR9] Cyr M, Riediger N (2021). (Re)claiming our bodies using a Two-Eyed Seeing approach: Health-At-Every-Size (HAES®) and Indigenous knowledge. Canadian Journal of Public Health.

[CR10] Department of Finance Tax Administration Division, Government of Newfoundland and Labrador. (2022). *Sugar Sweetened Beverage Tax*. https://www.gov.nl.ca/fin/files/Information-Circular-Sugar-Sweetened-Beverage-Tax-Sept-2022.pdf. Accessed Dec 2022.

[CR11] Eisenhauer E (2001). In poor health: Supermarket redlining and urban nutrition. GeoJournal.

[CR12] Gripper AB, Nethery R, Cowger TL, White M, Kawachi I, Adamkiewicz G (2022). Community solutions to food apartheid: A spatial analysis of community food-growing spaces and neighborhood demographics in Philadelphia. Social Science & Medicine.

[CR13] Health Canada. (2017). Reference guide to understanding and using the data: 2015 Canadian Community Health Survey-Nutrition. https://www.canada.ca/content/dam/hc-sc/documents/services/food-nutrition/food-nutrition-surveillance/ReferenceGuide2015CCHS-Nutr_Eng_Final_06192017.pdf. Accessed Dec 2022.

[CR14] Jones, A. C., Kirkpatrick, S. I., & Hammond, D. (2019). Beverage consumption and energy intake among Canadians: Analyses of 2004 and 2015 national dietary intake data. *Nutrition Journal, 18*(60), 1–14. https://doi-org.uml.idm.oclc.org/10.1186/s12937-019-0488-5 10.1186/s12937-019-0488-5 PMC6800499 31627756

[CR15] Kelder SH, Perry CL, Klepp KI, Lytle LL (1994). Longitudinal tracking of adolescent smoking, physical activity, and food choice behaviors. American Journal of Public Health.

[CR16] Malik VS, Popkin BM, Bray GA, Després JP, Willett WC, Hu FB (2010). Sugar-sweetened beverages and risk of metabolic syndrome and type 2 diabetes: A meta-analysis. Diabetes Care.

[CR17] Minkler, M., & Wallerstein, N. (Eds.). (2008). Community-based participatory research for health: From process to outcomes. John Wiley & Sons.

[CR18] Manitoba Collaborative Data Portal. (2021). *Winnipeg Food Atlas.* Manitoba Collaborative Data Portal. http://www.mbcdp.ca/fns.html. Accessed Dec 2022.

[CR19] Pittman C (2020). “Shopping while Black”: Black consumers’ management of racial stigma and racial profiling in retail settings. Journal of Consumer Culture.

[CR20] Poudrier J (2007). The geneticization of Aboriginal diabetes and obesity: Adding another scene to the story of the thrifty gene. Canadian Review of Sociology and Anthropology.

[CR21] Riediger, N. D., LaPlante, J., Mudryj, A., & Clair, L. (2022). Examining differences in diet quality between Canadian Indigenous and non-Indigenous adults: Results from the 2004 and 2015 Canadian Community Health Survey Nutrition Surveys. *Canadian Journal of Public Health*, *113*, 374–384. https://doi-org.uml.idm.oclc.org/10.17269/s41997-021-00580-x 10.17269/s41997-021-00580-x PMC9043166 35015285

[CR22] Saldaña J (2011). Fundamentals of qualitative research.

[CR23] Skinner E, Masuda JR (2013). Right to a healthy city? Examining the relationship between urban space and health inequity by Aboriginal youth artist-activists in Winnipeg. Social Science & Medicine.

[CR24] Smith, L. T. (2021). Decolonizing methodologies: Research and indigenous peoples. Bloomsbury Publishing.

[CR25] Tarasuk, V., Mitchell, A., & Dachner, N. (2014). Household food insecurity in Canada, 2012. Toronto, ON: Research to identify policy options to reduce food insecurity (PROOF). https://proof.utoronto.ca/resources/proof-annual-reports/annual-report-2012/

[CR26] Veugelers PJ, Taylor JP, Ohinmaa A, Liu S, Munasinghe LL, Maximova K (2022). To tax or not to tax? That’s the sugar-coated question. Canadian Journal of Public Health.

[CR27] Vilar-Compte M, Burrola-Méndez S, Lozano-Marrufo A, Ferré-Eguiluz I, Flores D, Gaitán-Rossi P, Teruel G, Pérez-Escamilla R (2021). Urban poverty and nutrition challenges associated with accessibility to a healthy diet: A global systematic literature review. International Journal for Equity in Health.

[CR28] Warren, C., Hobin, E., Manuel, D. G., Anderson, L. N., Hammond, D., Jessri, M., Arcand, J., L’Abbé, M., Li, Y., Rosella, L. C., Manson, H. & Smith, B. T. (2022). Socioeconomic position and consumption of sugary drinks, sugar-sweetened beverages and 100% juice among Canadians: A cross-sectional analysis of the 2015 Canadian Community Health Survey–Nutrition. *Canadian Journal of Public Health, 113*(3), 341–362. https://doi-org.uml.idm.oclc.org/10.17269/s41997-021-00602-8 10.17269/s41997-021-00602-8 PMC9043056 35138596

[CR29] Waugh, A. (2022). Exploring the acceptability of sugar-sweetened beverage taxes amongst residents of River Heights, Winnipeg: A critical discourse analysis. [Master’s thesis, University of Manitoba]. MSpace. http://hdl.handle.net/1993/36863

[CR30] Winnipeg Regional Health Authority (WRHA). (2015). *Point Douglas: Community area profile*. Winnipeg, MB: Evaluation Platform. https://wrha.mb.ca/files/cha-2014-profile-point-douglas.pdf. Accessed Nov 2022.

[CR31] Winnipeg Regional Health Authority (WRHA). (2019). *Point Douglas: Community area profile*. Winnipeg, MB: Evaluation Platform. https://wrha.mb.ca/files/cha-2019-profile-point-douglas.pdf. Accessed Nov 2022.

[CR32] Zenk SN, Schulz AJ, Israel BA, Mentz G, Miranda PY, Opperman A, Odoms-Young AM (2014). Food shopping behaviours and exposure to discrimination. Public Health Nutrition.

